# Supplementary material for: HantaNet: A New MicrobeTrace Application for Hantavirus Classification, Genomic Surveillance, Epidemiology and Outbreak Investigations
Source: Viruses. 2023 Nov 2;15(11):2208. doi: 10.3390/v15112208 (PMC10675615; doi:10.3390/v15112208)
Supplement: Supplementary file 1 [file viruses-15-02208-s001.zip › Supplementary_Materials_Proofread/Supplementary_Figures_Proofread.docx]

**Supplementary Figures**

**
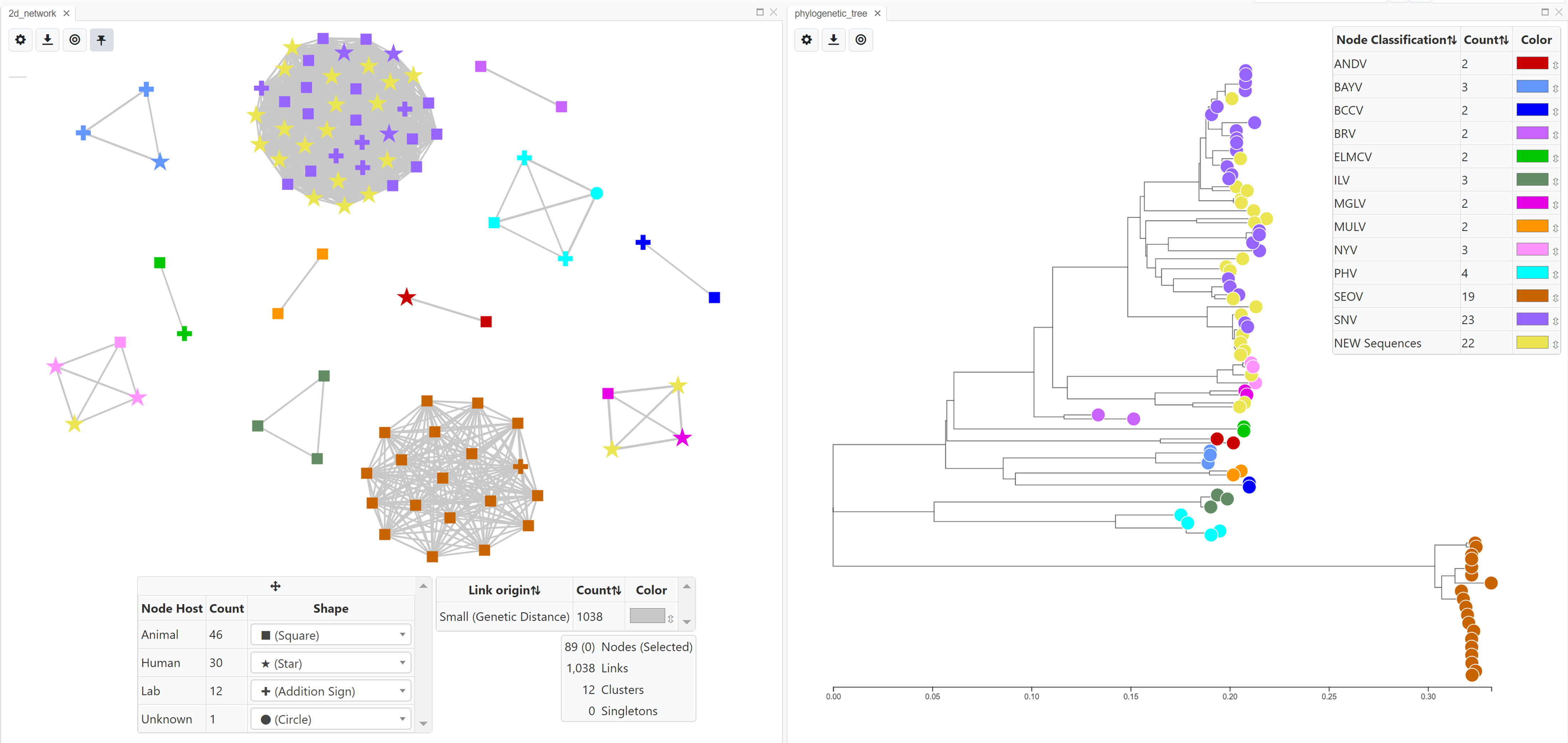
**

**Figure S1.** HantaNet dashboard built with the 2D network and unrooted phylogenetic tree views in the small (S, nucleoprotein) reference gene segment module. The TN93 nucleotide substitution model was applied at a 0.124 substitutions per site genetic distance cutoff to obtain hantavirus strain-specific clusters (n = 12) in the 2D network (**left**). Nodes represent individual sequences and were colored based on hantavirus classification as shown in the key table next to the phylogenetic tree (**right**). Nodes were shaped by animal (square), human (star) or unknown (circle) host, and lab or viral isolate (addition sign).


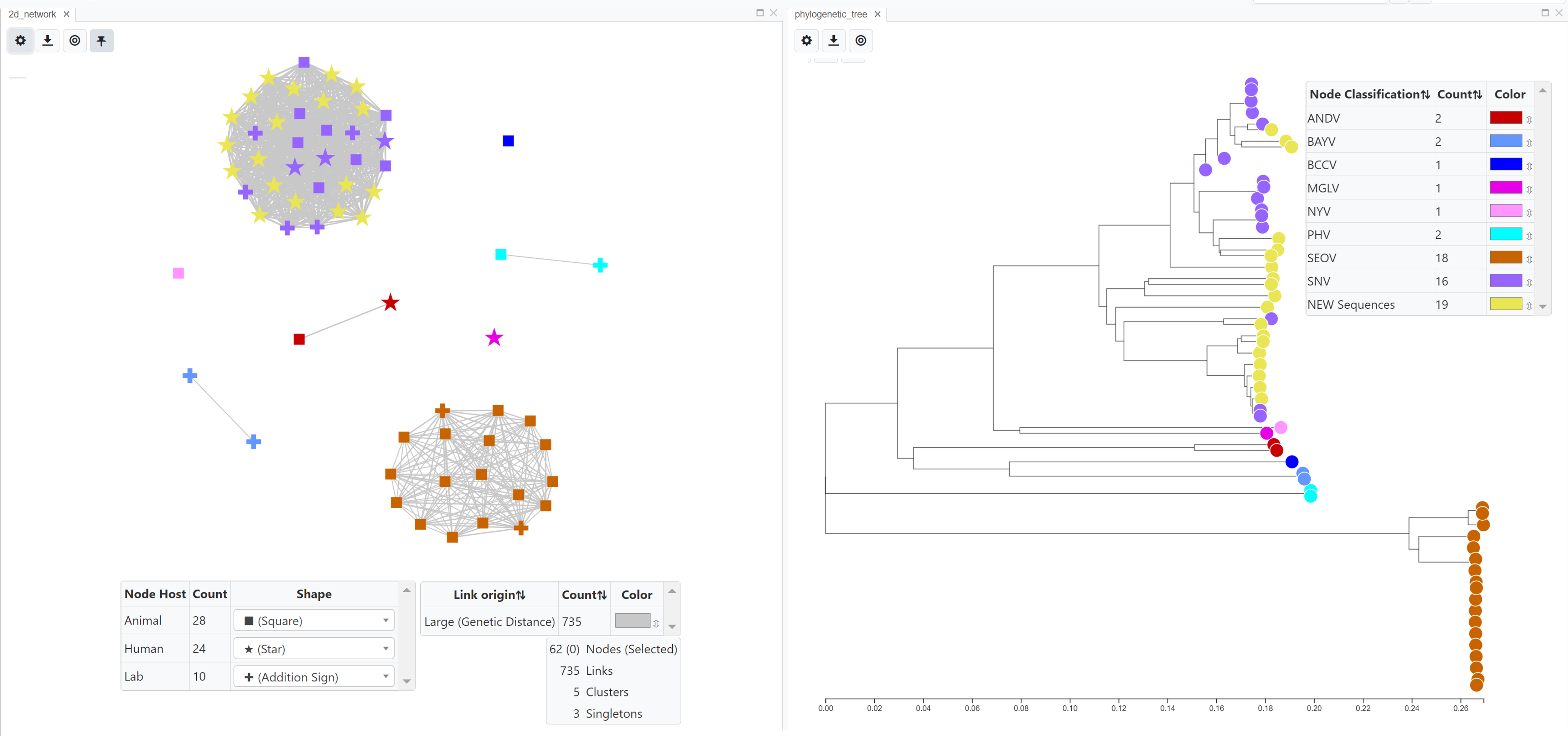


**Figure S2.** HantaNet dashboard built with the 2D network and unrooted phylogenetic tree views in the large (L, RNA polymerase) reference gene segment module. The TN93 nucleotide substitution model was applied at a 0.144 substitutions per site genetic distance cutoff to obtain hantavirus strain-specific clusters (n = 8; 5 clusters of two or more sequences and three singletons) in the 2D network (**left**). Nodes represent individual sequences and were colored based on hantavirus classification as shown in the key table next to the phylogenetic tree (**right**). Nodes were shaped by animal (square) or human (star) host, and lab or viral isolate (addition sign).

**Figure S3.** HantaNet dashboard built with the 2D network and unrooted phylogenetic tree views in the medium (M, glycoprotein) reference gene segment module. We previously loaded a FASTA alignment with M reference and test sequences in HantaNet to build a network and phylogenetic tree (Figure 2). We used this dataset to build an M phylogenetic tree with the MEGA software (not shown) to test loading a Newick tree and building the network in HantaNet. (A) The Newick tree was loaded into the HantaNet M module, which calculates the patristic distances as shown by the blue genetic links in the 2D network (**left**) and the branches in the phylogenetic tree view (**right**). The network (**left**) was composed of 9 clusters of two or more sequences and three singletons. The dashed lines represent the genetic link overlay of the pairwise genetic distances calculated from the reference FASTA alignments (gray links) and the patristic tree distances (blue links). Nodes represent individual sequences and were colored based on hantavirus classification as shown in the key table next to the phylogenetic tree (**right**). We found that three new sequences (yellow nodes with orange borders) did not cluster with any reference at the default genetic distance cutoff of 0.2 substitutions per site in the network (**left**). These sequences clustered with Monongahela virus (MGLV) (n = 2; magenta nodes) or New York virus (NYV) (n = 1; pink nodes) references. (B) Hence, we increased the genetic distance cutoff to identify their closest genetic relatives in the network (**left**). At genetic distance of 0.261 substitutions per site, we found that the three new sequences linked to MGLV and NYV references, which belonged to a bigger cluster that also included Sin Nombre virus (SNV) and Blue River virus (BRV) sequences. The network was then updated to include 6 clusters of two or more sequences and one singleton. Nodes were shaped by host: animal (square), human (star), lab or viral isolate (addition sign), and null (wye).
