# Supplementary figures and images for: HantaNet: A New MicrobeTrace Application for Hantavirus Classification, Genomic Surveillance, Epidemiology and Outbreak Investigations
Source: Viruses. 2023 Nov 2;15(11):2208. doi: 10.3390/v15112208 (PMC10675615; doi:10.3390/v15112208)

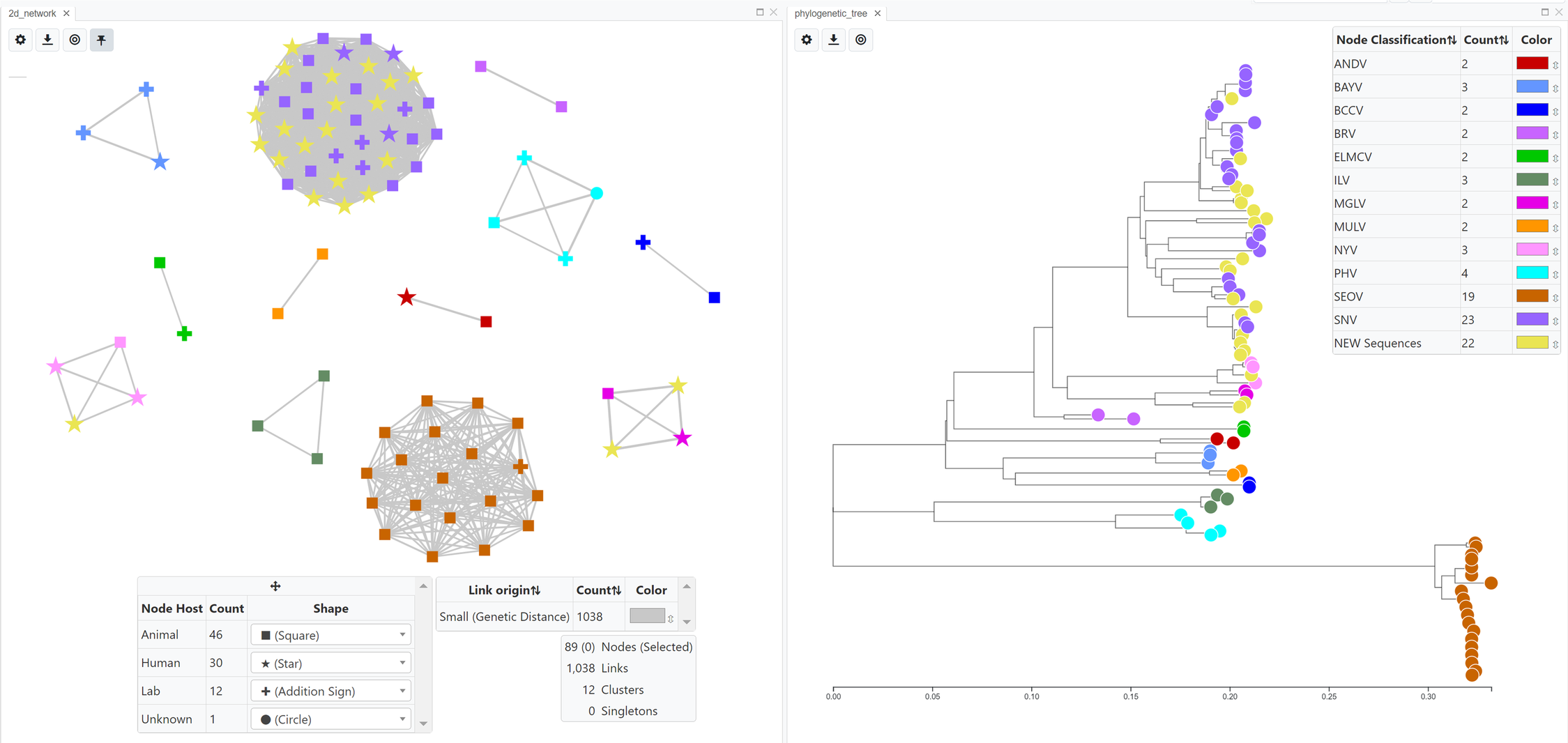

Supplement: Supplementary file 1 [file viruses-15-02208-s001.zip › Supplementary_Materials_Proofread/Figure S1.tif]

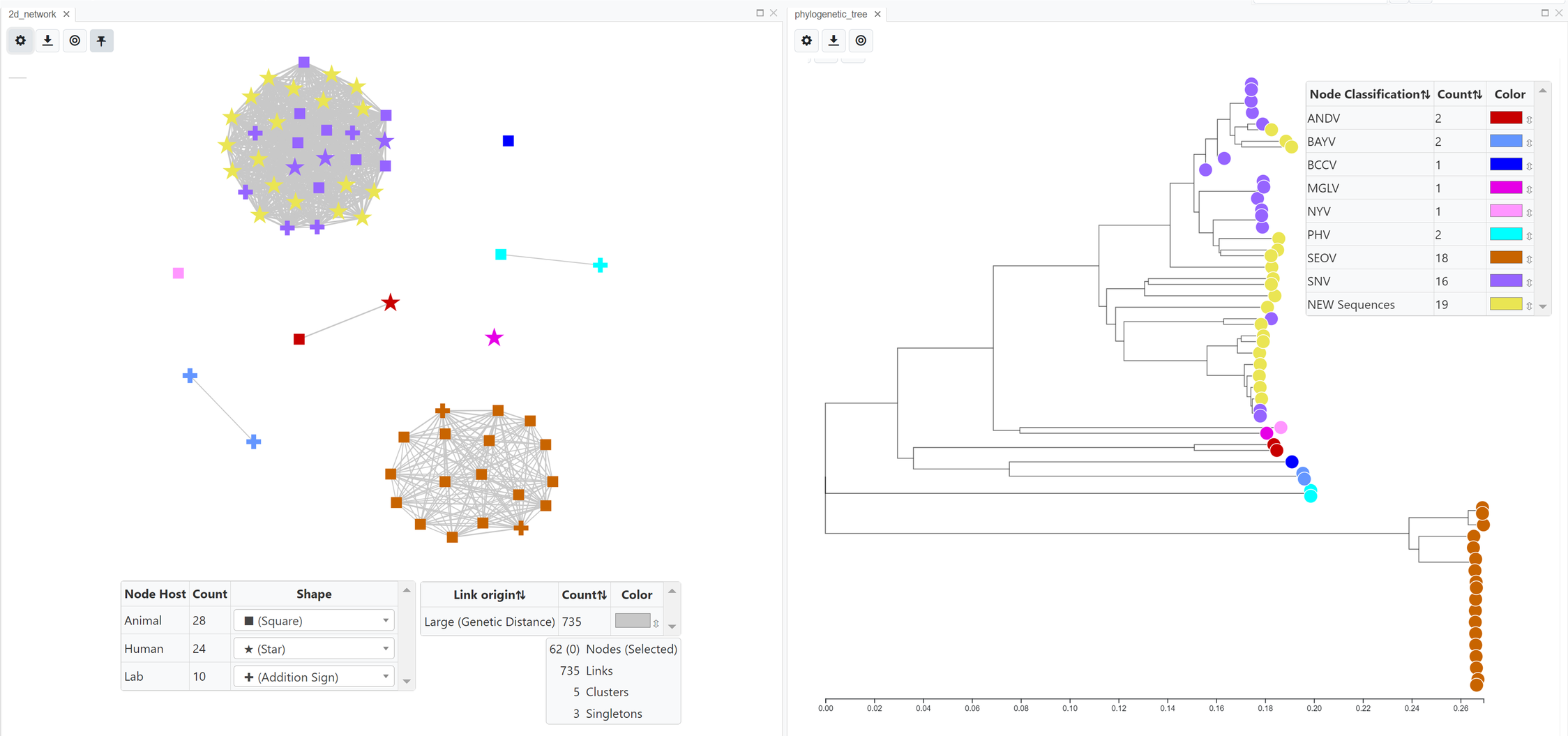

Supplement: Supplementary file 1 [file viruses-15-02208-s001.zip › Supplementary_Materials_Proofread/Figure S2.tif]

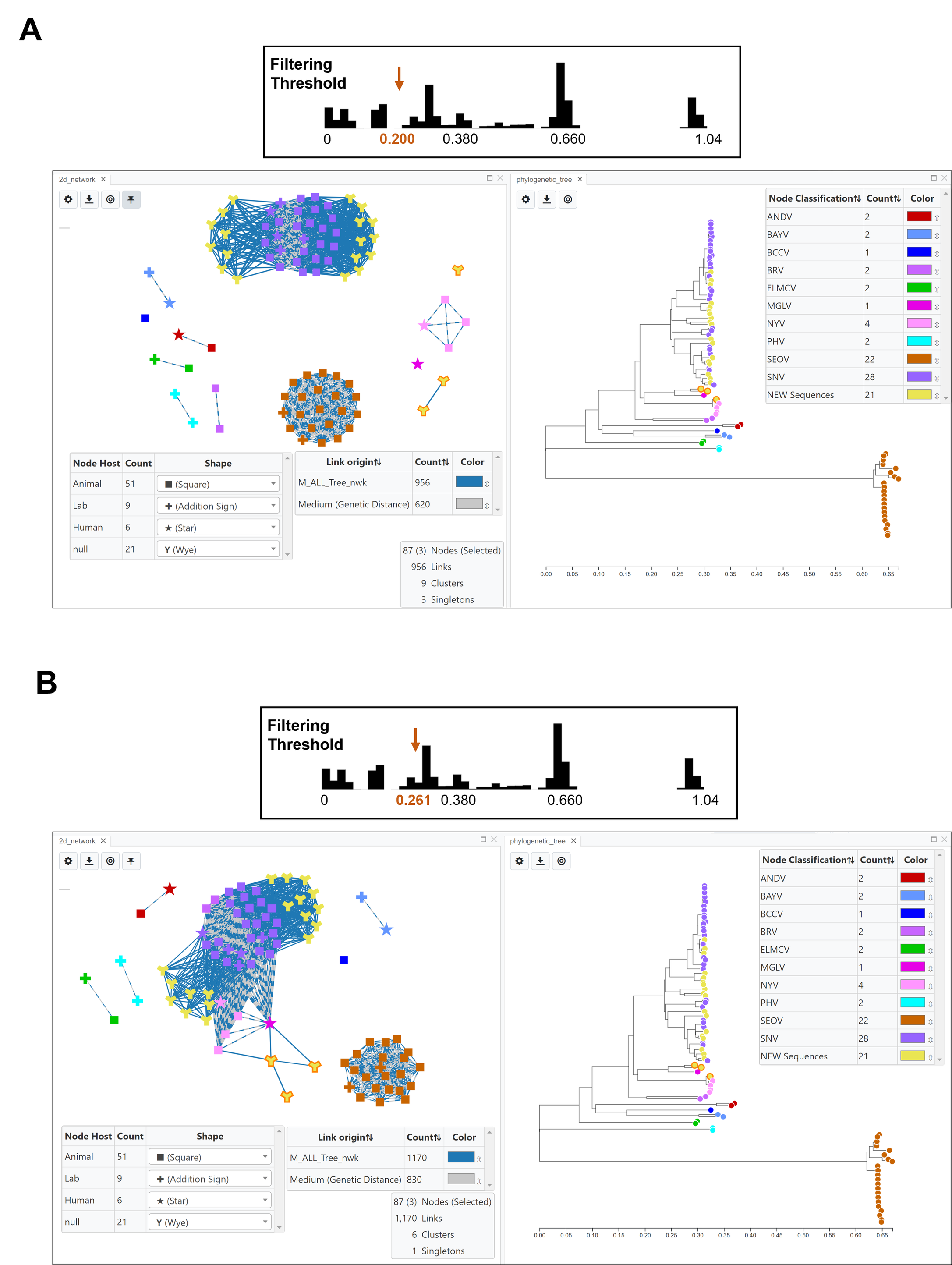

Supplement: Supplementary file 1 [file viruses-15-02208-s001.zip › Supplementary_Materials_Proofread/Figure S3.tif]
